# *C. elegans* survival assays to discern global and transcription-coupled nucleotide excision repair

**DOI:** 10.1016/j.xpro.2021.100586

**Published:** 2021-06-08

**Authors:** Melanie van der Woude, Hannes Lans

**Affiliations:** 1 Department of Molecular Genetics, Erasmus MC Cancer Institute, Erasmus University Medical Center, 3000 CA Rotterdam, the Netherlands

**Keywords:** Cell biology, Genetics, Model organisms, Molecular biology

## Abstract

Global genome nucleotide excision repair (GG-NER) and transcription-coupled nucleotide excision repair (TC-NER) protect cells against a variety of helix-distorting DNA lesions. In *C. elegans*, GG-NER primarily acts in proliferative germ cells and embryos, while TC-NER acts in post-mitotic somatic cells to maintain transcription. We leverage this difference to distinguish whether proteins function in GG-NER and/or TC-NER by straightforward UV survival assays. Here, we detail a protocol for these assays, using GG-NER factor *xpc-1* and TC-NER factor *csb-1* as examples.

For complete details on the use and execution of this protocol, please refer to Sabatella et al. (2021).

## Highlights

• Robust assays to evaluate protein involvement in nucleotide excision repair
• Survival assays to discern global genome from transcription-coupled repair
• *C. elegans* as an additive *in vivo* DNA damage response model

## Graphical abstract

**Figure:**
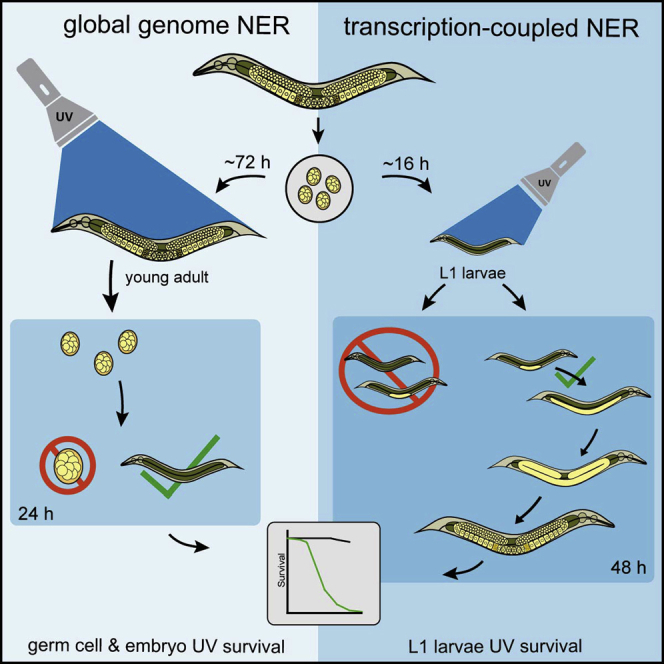

## Before you begin

In order to perform UV survival assays with *Caenorhabditis elegans,* the animal needs to be cultured and maintained. We therefore first describe basic culturing and maintenance of this animal. *C. elegans* is commonly maintained on Nematode Growth Medium (NGM) agar plates using *Escherichia coli* strain OP50 as a food source (Brenner, 1974). In our survival assays, we also use HT115(DE3) *E. coli* as food source, as these bacteria form a thinner lawn than OP50 and therefore block less UV light (Lans et al., 2010). Additional information about *C. elegans* as model organism, its biology and laboratory techniques can be found on the online resource www.wormbook.org (Girard et al., 2007; Stiernagle, 2006). It is important to feed *C. elegans* ample food before the survival assays start, such that the animals are minimally stressed. Each survival assay needs to start with sufficient culture plates containing well-fed adult animals. Table 1 lists how many culture plates and assay plates should be prepared for each experiment.

**Table 1:** Amount of bacterial liquid culture and number of NGM plates used in the protocol

	Germ cell and embryo UV survival		L1 larvae UV survival	
	Per tested strain	Example Figure 2	Per tested strain	Example Figure 2
OP50 *E. coli* (μL)	3150	9450	N/A	N/A
HT115(DE3) *E. coli* (μL)	625	1875	625	1875
90 mm unseeded NGM plates	5	15	N/A	N/A
90 mm OP50 NGM plates	9	27	N/A	N/A
60 mm HT115(DE3) NGM plates	25	75	25	75

Please note that these numbers only include the plates needed for the assay itself and not for culturing of *C. elegans*.

### NGM culture plate preparation

**Timing: 2–3 days**

*C. elegans* is simply grown on NGM agar plates containing bacteria as food. When, upon visual inspection, food becomes limiting, animals need to be transferred to a new plate, which can be done by picking animals individually with a worm pick or collectively by cutting out and transferring an agar slab with animals. NGM culture plate preparation for 1 L medium, which equals ∼ 45× 90 mm or ∼120× 60 mm plates, is described below.

1. Weigh 3 g NaCl, 7.5 g casein digest and 17 g agar in a 2 liter autoclavable glass bottle and add 1000 mL double distilled water (ddH_2_O).
2. Sterilize by autoclaving for 20 min at 121°C.

**CRITICAL:** Make sure the cap is loosened prior to autoclaving as a closed cap could cause the bottle to explode due to pressure build up.

3. Air-cool the autoclaved bottle to 55°C. Next, shake the bottle to create a homogeneous mixture.
4. Add 25 mL KPO_4_ buffer (1 M stock solution, pH 6.0, see table in Materials), 1 mL MgSO_4_ (1 M stock solution), 1 mL CaCl_2_ (1 M stock solution), and 1 mL of 5 mg/mL cholesterol (see table in Materials).

**CRITICAL:** The KPO_4_ buffer and MgSO_4_ and CaCl_2_ solutions must also be sterilized by autoclaving for 20 min and air-cooled to 20°C–22°C before use. This is not necessary for the cholesterol solution, as this is dissolved in ethanol and therefore already sterile.

***Optional:*** To avoid contaminations, an antibiotic can be added to the NGM, in which case modified OP50 or HT115(DE3) bacteria (ampicillin resistant) should be used as food source (see bacterial food source). Note that *C. elegans* itself is sensitive to certain antibiotics, such as puromycin (Semple et al., 2010) or G418 (Giordano-Santini et al., 2010), which therefore cannot be used.

***Optional:*** Instead of air-cooling the autoclaved bottle, a water bath or incubator can be used to cool and keep bottles at 55°C. This is especially convenient if multiple bottles are used simultaneously.

5. Dispense the NGM into either 90 mm or 60 mm Petri dishes, as needed, using a peristaltic pump. Use approximately 10 mL NGM per 90 mm and 5 mL NGM per 60 mm dish.

***Alternatives:*** If a peristaltic pump is not available, the NGM can also be poured by hand into the plates using a glass flask equipped with a pouring ring (which avoids spilling).

6. Let the NGM in the plates solidify at 20°C–22°C for 2–3 days before use, to allow detection of contaminants and to allow excess moisture to evaporate.
7. If used immediately, store the plates upside down at 20°C–22°C. For longer term storage (up to 3 weeks), store the plates upside down at 4°C.

**CRITICAL:** The NGM plates should not dry out, which can be avoided by using an air-tight container for storage.

### Bacterial food source

**Timing: 3–4 days**

NGM plates are seeded with bacteria. We use OP50 *E. coli* as food source for culturing on 90 mm plates and HT115(DE3) *E. coli* as food source for the UV survival assays on 60 mm plates.

8. Grow OP50 and HT115(DE3) *E. coli* as single colonies on LB agar plates (See table in Materials) at 37°C for 14–16 h.
9. Pick a single OP50 or HT115(DE3) *E. coli* colony and prepare a liquid culture by inoculating the colony into LB medium (see table in Materials) and culturing while shaking vigorously, at 37°C for 14–16 h.

***Note:*** The amount of liquid culture depends on the number of plates that need to be seeded for the experiments. In general, a liquid bacterial culture of 20 mL is sufficient for 50 × 90 mm NGM plates seeded with 350 μl or 750 × 60 mm plates seeded with 25 μl bacterial culture. To achieve maximum reproducibility, it is better to always grow bacteria in the same volume and to grow multiple cultures of this volume if more bacteria are needed. Typically, we grow bacteria to maximum density in the LB medium, i.e., OD600 > 0.9.

10. Dispense, under semi-sterile conditions, 350 μl of OP50 *E. coli* liquid culture to each of the 90 mm dishes, and spread the bacteria over the whole NGM culture plate using a bacterial cell spreader. Allow the resulting bacterial lawn to grow for 12–16 h at 20°C–22°C.

***Alternatives:*** Instead of a bacterial cell spreader, a glass pipet bend into a spreader can be used.

***Alternatives:*** Instead of LB medium, it is possible to use any other standard rich growth medium to culture the bacteria.

**CRITICAL:** Avoid extending the bacterial lawn too much to the edges of the NGM plate as *C. elegans* may crawl up the sides of the dish where they dry out and die.

11. Dispense, under semi-sterile conditions, 25 μl of HT115(DE3) *E. coli* liquid culture to the middle of each of the 60 mm dishes. These bacteria should not be spread and only shortly grown, for 4–6 h at 20°C–22°C, to ensure that only a thin bacterial lawn is formed.

**CRITICAL:** The 60 mm HT115(DE3) *E. coli* containing plates should not be stored at 20°C–22°C for longer than 6 h to avoid too much bacterial growth, which could shield *C. elegans* from UV light and make it more difficult to score eggs and small early larval stages under the microscope.

12. All plates can be stored upside down at 4°C for at least several weeks.

**CRITICAL:** The bacterial plates should not dry out, which can be avoided by using an air-tight container for storage.

## Key resources table

**Table:** 

REAGENT or RESOURCE	SOURCE	IDENTIFIER
**Bacterial and viral strains**		

*Escherichia coli*, OP50	Caenorhabditis Genetics Center	OP50
*Escherichia coli*, HT115(DE3)	Caenorhabditis Genetics Center	HT115(DE3)

**Chemicals, peptides, and recombinant proteins**		

Sodium chloride (NaCl)	Sigma-Aldrich	S9888
Bacto agar	Difco (Thermo Fisher)	214030
Casein digest	Difco (Thermo Fisher)	211610
Magnesium sulfate (MgSO_4_)	Sigma-Aldrich	M2643
Cholesterol	Sigma-Aldrich	C3045-5G
Ethanol (EtOH)	TechniSolv	200-578-6
Monopotassium phosphate (KH_2_PO_4_)	Sigma-Aldrich	71504-1KG
Dipotassium phosphate (K_2_HPO_4_)	Honeywell	778-77-0
LB Broth (Lennox)	Sigma-Aldrich	L7658-1KG
Sodium hydroxide (NaOH)	Sigma-Aldrich	480878
Sodium hypochlorite, 10%–15% active chlorine (NaClO)	Acros Organics	7681-52-9
Sodium phophate dibasic dihydrate (Na_2_HPO_4_)	Fluka	71645-1KG
Kanamycin	Sigma-Aldrich	K0254
Ampicillin	Sigma-Aldrich	A1593

**Experimental models: organisms/strains**		

*C. elegans* wild-type N2	Caenorhabditis Genetics Center	Bristol N2
*C. elegans xpc-1(tm3886)*	National Bioresource Project for the nematode	*xpc-1(tm3886)*
*C. elegans csb-1(ok2335)*	Caenorhabditis Genetics Center	RB1801

**Software and algorithms**		

Windows Excel	N/A	Microsoft Excel, RRID:SCR_016137
GraphPad Prism v8	GraphPad Software, Inc	https://www.graphpad.com:443/, RRID:SCR_002798

**Other**		

Petri plates, 90 mm	VWR	391-0601
Petri plates, 60 mm	Falcon	353004
SZX10 stereomicroscope	Olympus	SZX10
UV dosimeter (UVX radiometer)	UVP	UVX-31
UVB lamp TL 40 W/12	Philips	928011301230
Spectrophotometer	Thermo Scientific	Genesys 20

## Materials and equipment

**Table:** LB agar plates

Reagent	Final concentration	Amount
LB Broth	N/A	10.3 g
Agar	N/A	7.5 g
ddH_2_O	N/A	500 mL
Optional		
Kanamycin	50 μg/mL	N/A
Ampicilin	50 μg/mL	N/A

Use after high pressure autoclaving. Cool to 55°C–60°C. Add antibiotic of choice. Dispense into 90 mm petri dishes and air-dry the plats at 20°C–22°C. Store at 4°C for up to 2 weeks.

**Table:** KPO_4_ buffer

Reagent	Final concentration	Amount
KH_2_PO_4_	N/A	108.3 g
K_2_HPO_4_	N/A	35.6 g
ddH_2_O	N/A	1 L

Adjust the pH to 6.0. After checking pH sterilize using high pressure autoclaving. Store at 20°C–22°C for at least 1 year.

**Table:** M9 buffer

Reagent	Final concentration	Amount
KH_2_PO_4_	N/A	3 g
Na_2_HPO_4_	N/A	6 g
NaCl	N/A	5 g
ddH_2_O	N/A	1 L
MgSO_4_	1 mM	1 mL
ddH_2_O	N/A	1 L

Use after high pressure autoclaving. Cool to 20°C–22°C and add 1 mL of MgSO_4_ (1 M) per liter. Store at 20°C–22°C for at least 1 year.

**Table:** Cholesterol

Reagent	Final concentration	Amount
Cholesterol	N/A	50 mg
Ethanol	N/A	10 mL

Store at −20°C for at least 1 year.

**Table:** Bleach / NaOH mixtureF

Reagent	Final concentration	Amount
NaOH (4 M)	1.6 M	500 μL
NaClO	60%	750 μL

***Note:*** This mixture should be made fresh at the day of use.

## Step-by-step method details

### Germ cell and embryo UV survival assay

In this assay, the viability of embryos of UV-irradiated adult animals is measured, by determining whether the eggs laid by the adult animals hatch or die. As *C. elegans* germ cells depend on GG-NER to overcome UV-induced DNA damage, embryonic survival in this assay reflects GG-NER capacity (Lans et al., 2010; Sabatella et al., 2021). The assay takes five days in total. Below, in steps 1 to 29, we describe in detail each consecutive step to be performed in chronological order. In a typical germ cell and embryo UV survival assay, we irradiate with 20, 40, 80 and 120 J/m^2^ UVB and include a non-irradiated control. Per strain, four OP50-seeded plates are needed for culturing of a synchronized population after which for each dose an unseeded 90 mm NGM plate is needed for irradiation and one OP50-seeded 90 mm NGM plate for recovery and five HT115(DE3)-seeded 60 mm plates for egg laying. Thus, per strain this is nine OP50 90 mm plates, five unseeded NGM 90 mm plates and 25 HT115(DE3) 60 mm plates. The number of plates used per experiment is listed in Table 1.

#### Day 1: Bleaching *C. elegans* to synchronize life cycle.

**Timing: 0.5–1 h**

1. Start with a single 90 mm NGM plate per strain, full of well fed, gravid adult animals. Wash off the worms with 2 mL of M9 buffer and transfer the worms to a 2 mL tube.
2. Pellet the worms by spinning down at 400 *g* for 2 min.

**CRITICAL:** If the supernatant is not clear, wash the worms by removing the supernatant and adding again 2 mL of M9 buffer and spinning down. Repeat this step until the supernatant is a clear solution. Bacteria often cause the solution to be troubled and could negatively affect the bleaching. Alternative to spinning down, adult worms can be pelleted by gravity.

3. Remove the supernatant until 500 μL of M9 buffer with worms per tube is left.

***Note:*** Supernatant can be removed by suction using a pipette or vacuum aspirator.

4. Prepare fresh NaClO/NaOH solution by mixing 750 μL NaClO with 500 μL NaOH (see materials and equipment).

***Note:*** NaOH (sodium hydroxide) is a strong base and therefore is very corrosive. Avoid contact with eyes and skin as it can cause blindness and irritation to the skin due to skin burns. Therefore, use eye protection and gloves.

***Note:*** NaClO (sodium hypochlorite) is corrosive and can causes severe skin burns and eye damage. Therefore, use eye protection and gloves.

5. Add 250 μL NaClO/NaOH to the 500 μL M9 buffer with worms. Vortex this mixture and wait until the adult worms dissolve and only the eggs remain, which can be checked under the microscope.

***Note:*** This step takes approximately 3–4 min, depending on e.g., the strength and freshness of the bleach solution. The egg shell protects embryos from dissolving, but if the treatment is too long or harsh, also the embryos will die.

6. Add 1 mL M9 buffer and mix thoroughly to slow down the bleaching reaction.
7. Spin at 400 *g* for 2 min to pellet the eggs.
8. Wash twice by removing the supernatant, adding 1 mL of M9 buffer and spinning down for 2 min.
9. Remove the supernatant and gently take up the eggs in 100 μL of M9 buffer. Resuspend thoroughly by tapping the side of the tube.
10. Plate the eggs unto four OP50 *E. coli* 90 mm NGM plates by pipetting 25 μL unto each plate.
11. Leave the plates shortly with the lid open for fluids to evaporate.
12. Incubate for 72 h on 20°C to allow the embryos to grow into young adults.

**CRITICAL:** Some gene mutations affect embryogenesis and/or development of *C. elegans*. Therefore, for each strain it must be determined by visual inspection if sufficient viable eggs are present on each plate and if the worms have developed into the desired young adult stage 72 h after bleaching. If insufficient viable eggs are present, this can be overcome by bleaching more than one NGM plate with gravid adults in step 1. If after 72 h the animals are too young, a longer incubation period should be used.

**CRITICAL:** At the end of the 72 h, there should be sufficient well-fed young hermaphrodite animals. If too many eggs per plate are plated, this could cause animals to become starved, which could negatively affect their survival. Starvation can be avoided by using more plates to plate the eggs and adding not more than several hundred eggs per plate in step 10.

#### Day 4: UV irradiation of young adult worms

**Timing: 1–2 h**

13. Collect the young adult worms from the four plates by washing the worms off each plate with 2 mL of M9 buffer and transferring the worms to a 15 mL tube.
14. Pellet the worms by spinning down at 400 *g* for 2 min.
15. Remove the supernatant. Add 1 mL M9 buffer and transfer the worms to a 2 mL tube.
16. Wash three times by adding 1 mL M9 buffer, spinning down at 400 *g* for 2 min and removing the supernatant.

**CRITICAL:** This step is necessary to remove any bacteria sticking to the worms. This is important as these bacteria could shield against the UVB irradiation, which then penetrates less into the worm.

***Alternatives:*** Spinning in a centrifuge is not necessary, especially if there are many animals, as by gravity the adult worms will also sink to form a pellet at the bottom of the tube.

17. After the last washing step, remove the supernatant and take up the pelleted worms in 100 μL M9 buffer.
18. Distribute the worms over five unseeded, i.e., without bacteria, 90 mm NGM plates.

***Optional:*** We use five plates as we typically UV irradiate with 4 doses (20, 40, 80, 120 J/m^2^ UV-B) and include a non-irradiated control. If more or less UV doses are used, adjust the number of plates accordingly.

19. Leave the plates with the lid open for the liquid to evaporate.
20. UVB irradiate the worms with the desired UVB dose by placing each of the plates without lid under the UVB lamp.

**CRITICAL:** Determine the exact dose (J/m^2^) per time unit of the UV lamp using a UV dosimeter. In case the UVB lamp needs to warm up, make sure it is already turned on. For reproducibility, be sure to always position each plate at the same spot under the UV lamp.

***Note:*** During UVB irradiation, it is very important to shield yourself from the UVB light, as it causes DNA damage. Shielding can easily be done by wearing protective clothes like a common lab coat to cover all the skin, face shield to protect the eyes and face, and gloves to protect the hands.

21. Wash off the irradiated worms from the empty NGM plates with 2 mL M9 and plate the animals on five 90 mm OP50 NGM plates.
22. Leave the plates with the lid open for the liquid to evaporate.
23. Let the irradiated animals recover for 16 – 24 h at 20°C.

#### Day 5: Egg laying

**Timing: 4–5 h**

24. Per UVB dose, pick five times three adult hermaphrodite worms and plate these on five 60 mm HT115(DE3) NGM plates. Thus, in total for five UV doses, 25 HT115(DE3) NGM plates are required per strain.
25. Let the adult worms lay eggs for a period of 3–4 h at 20°C.

***Note:*** During this incubation period, the adult worms will lay eggs, which ideally should be approximately 50 eggs. However, this number can vary between strains and is often reduced if a strain is UV hypersensitive. If too many eggs are being laid, counting will be tedious. This can be avoided by plating fewer adult animals per plate or by reducing the time allowed for egg laying. If too few eggs are laid, the results are not reliable. This can be avoided by plating more irradiated worms per 60 mm plate such that more eggs are laid.

26. Remove the adult worms from the plates by picking.
27. Incubate the plates at 20°C for 16 h such that any viable eggs have time to hatch.

#### Day 6: Counting

**Timing: 1–2 h**

28. Count for each plate the number of unhatched and hatched eggs, i.e., the number of unviable embryos and viable larvae (Figure 1).

**Figure 1:**
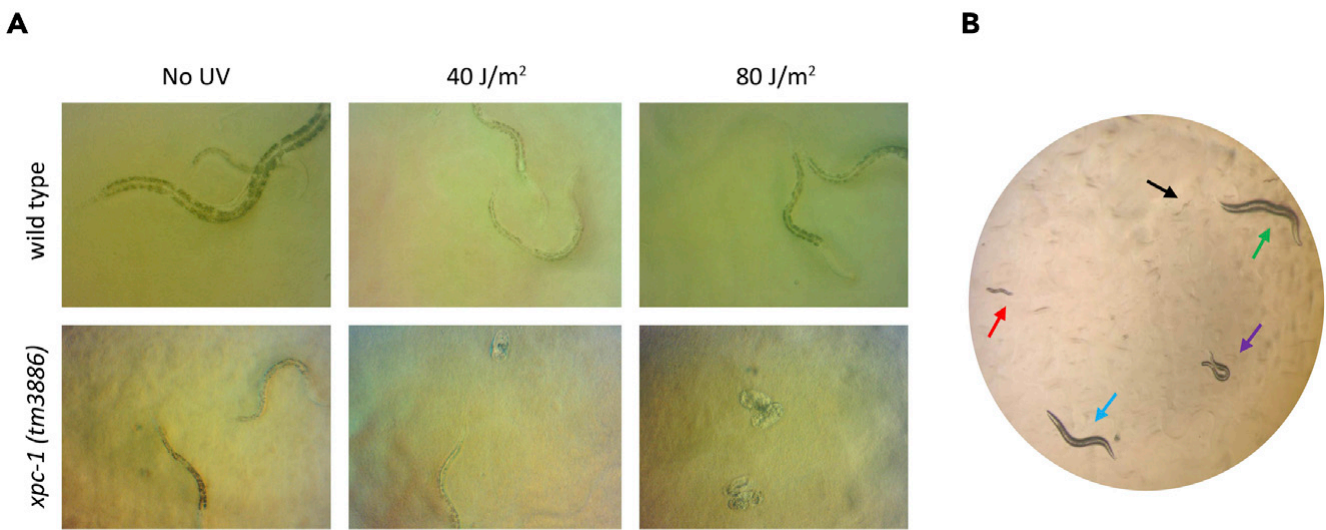
Stereomicroscopic views of *C. elegans* (A) Stereomicroscope view of *C. elegans* wild type and *xpc-1(tm3886)* mutants in the germ cell and embryo UV survival assay, exposed to no UV or to 40 or 80 J/m^2^ UVB. Eggs of *xpc-1* mutants fail to hatch with increasing UVB dose. (B) Stereomicroscope view of the different larval stages as counted in L1 larvae UV survival assay. L1 larvae are shown by black arrow, L2 larvae by red, L3 larvae by purple, L4 by blue and adult by green arrow.
29. The fraction or percentage UVB survival is calculated by dividing the number of hatched eggs by the total number of eggs and worms. This is first calculated for all individual plates, after which for each dose the mean survival of the five plates is determined.

### L1 larvae UV survival assay

In this assay, the development of larvae after UV irradiation is determined as measure of TC-NER capacity. TC-NER is activated during transcription when RNA polymerase II is blocked by helix-distorting lesions such as those induced by UV irradiation (Lans et al., 2019). When lesions cannot be repaired, i.e., if TC-NER is deficient, transcription is impaired, which results in arrested development of the irradiated L1 larvae (Bianco and Schumacher, 2018; Sabatella et al., 2021). As this can be easily scored by visual inspection, in this protocol we describe how the involvement of a gene of interest in TC-NER can be tested.

The assay takes four days in total. Below, in steps 30 to 37, we describe in detail each consecutive step to be performed in chronological order. Typically, in the L1 larvae UV survival assay we irradiate with 20, 40, 80 and 120 J/m^2^ UVB and include a non-irradiated control. Per strain, for each UVB dose five HT115(DE3) 60 mm NGM plates are needed. Thus, per strain this is 25 HT115(DE3) 60 mm plates (Table 1).

#### Day 1: Bleaching *C. elegans* plates to synchronize life cycle.

**Timing: 0.5–1 h**

30. Start with a single 90 mm NGM plate per strain, full of well fed, gravid adult worms. Perform steps 1 to 9 of the bleaching protocol as described above in the germ cell and embryo UV survival assay.

***Note:*** The bleaching procedure is similar as described above. For L1 larvae UV survival bleaching should be performed at the end of the day. Hatching of the eggs into the first larval stage takes place around 16–17 h post bleaching. Therefore, the bleaching of the worms in this L1 larvae UV survival assay should be performed 16–17 h prior to UV irradiation.

31. Dispense approximately 50–100 eggs, by checking under the microscope, on a 60 mm HT115(DE3) NGM plate. Add the drop just at the edge outside the bacterial lawn, so that hatched larvae can easily crawl into the food. Repeat this for five plates per UV dose per strain. This will be in total 25 plates per strain.

**CRITICAL:** Please be aware that the eggs should be dropped on the same spot on each plate, such that variations in bacterial layer are kept minimal, as bacteria around the worm can act as a protective shield from UV.

32. Leave the eggs to hatch at 20°C for 16–17 h.

***Alternatives:*** Instead of letting eggs hatch on HT115(DE3) 60 mm NGM plates, eggs can also be allowed to hatch in M9 buffer (Babu and Schumacher, 2016). This will provide a better way of synchronization, as starved L1 animals arrest development. Although we have never tested this experimentally, it should be considered that the lack of food may also stress the animals and therefore negatively affect survival.

#### Day 2: UV irradiation of L1 larvae

**Timing: 0.5–1 h**

33. Check under the microscope if the eggs have hatched into L1 larvae.

***Note:*** Some gene mutations affect hatching and/or development of *C. elegans*, because of which not all eggs may have hatched after 16–17 h. Therefore, plates should be visually inspected to determine whether eggs of each strain have hatched into L1 larvae.

34. UVB irradiate the L1 larvae with the desired UVB dose by placing each of the 60 mm HT115(DE3) plates without lid under the UVB lamp.

**CRITICAL:** Determine the exact dose (J/m^2^) per time unit of the UV lamp using a UV dosimeter. In case the UVB lamp needs to warm up, make sure the lamp is already turned on. For reproducibility, be sure to always position each plate at the same spot under the UV lamp.

***Note:*** During UVB irradiation, it is very important to shield yourself from the UVB light, as it causes DNA damage. Shielding can easily be done by wearing protective clothes like a common lab coat to cover all the skin, face shield to protect the eyes and face, and gloves to protect the hands.

35. Leave the L1 larvae to develop into subsequent larval stages at 20°C for 2 days.

#### Day 4: Counting

**Timing: 1–2 h**

36. For each plate, count the number of developmentally arrested L1 and L2 larvae and the number of non-arrested viable L3, L4 and young adult animals. (Figure 1B).

***Alternatives:*** Counting the number of different larvae can also be performed automatically using a flow cytometer designed for worms (Babu and Schumacher, 2016).

37. The fraction or percentage UVB survival is calculated by dividing the number of arrested L1/L2 larvae by the total number of all larval stages and adult worms. This is first calculated for all individual plates, after which for each dose the mean survival of the five plates is determined.

## Expected outcomes

**Figure 2:**
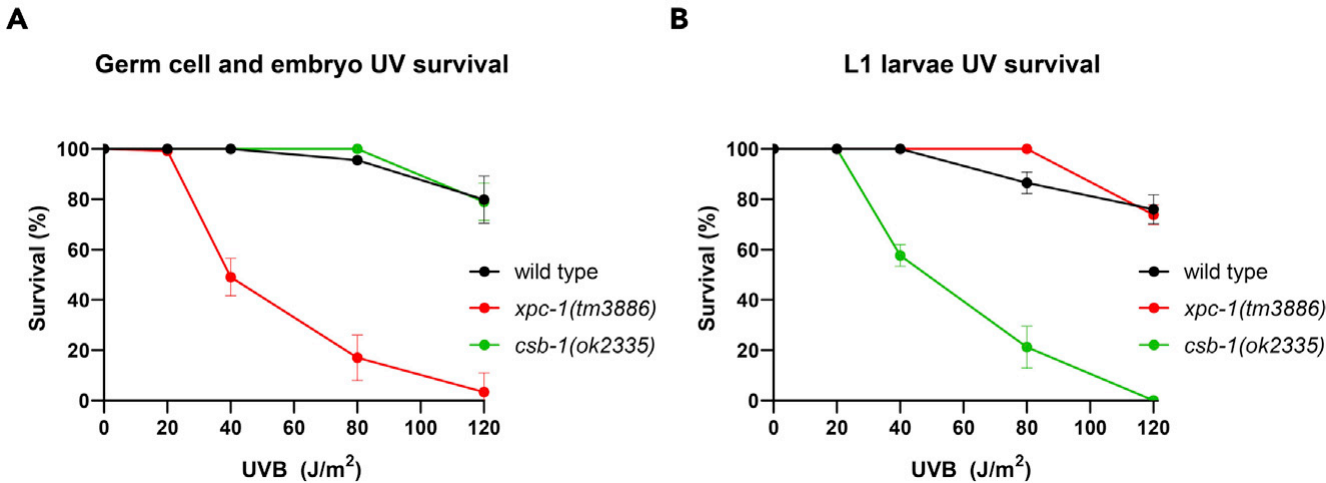
GG-NER and TC-NER UV survival assays Graphs of a representative (A) germ cell and embryo UV survival and (B) L1 larvae UV survival experiment with wild type, *xpc-1(tm3886)* and *csb-1(ok2335)* animals. Each data point represents the average of five plates. Error bars depict s.d. values.

A GG-NER-deficient strain will show reduced UV survival in the germ cell and embryo UV survival assay, while a TC-NER deficient strain will show slower or arrested larval development due to UV-induced DNA damage in the L1 larvae assay. Indeed, we observed that *xpc-1(tm3886)* mutant animals, which lack the function of GG-NER-initiation factor XPC-1, are UV hypersensitive in the germ cell and embryo UV survival assay, but *csb-1(ok2335)* mutant animals are not (Figures 1 and 2A). In contrast, *csb-1* animals, which lack the function of TC-NER initiation factor CSB-1, show reduced UV survival in the L1 larvae UV survival assay, whereas *xpc-1* mutants did not (Figure 2B).

*C. elegans* mutants for many different genes are available from the *C. elegans* Deletion Mutant Consortium (C. elegans Deletion Mutant Consortium, 2012) and gene knockdown or knockout can be achieved relatively easily using a variety of methods (Nance and Frøkjær-Jensen, 2019). Therefore, the protocols that we show here and have used in previous studies (Lans et al., 2013, 2010; Sabatella et al., 2021) are easy tools to study if a novel factor is essential for NER in the entire genome or during active transcription or both.

## Quantification and statistical analysis

Typically, each of the survival experiments should be repeated at least three times to ensure reproducibility of the results. Statistical significance can be determined using a standard t test, if two strains are compared, or one-way ANOVA, it multiple strains are compared.

## Limitations

Overall, we find that the described survival assays provide robust and reproducible results and are therefore very useful in identifying genes that are involved in GG- and/or TC-NER. Most DNA damage response pathways are conserved in *C. elegans*, in particular also NER (Boulton et al., 2002; Lans and Vermeulen, 2015, 2011), making these assays also useful for researchers using other model organisms such as mice or yeast. However, lack of evolutionary conservation could be a limitation in applying the techniques.

In our experience, a UVB lamp (wavelength ∼300 nm) is most reliable as UV source to damage DNA to test NER capacity, as UVB penetrates best through the multiple cell layers of *C. elegans* (Lans et al., 2010). Standard UVB TL tubes in standard fittings can be used or, as in our case, in a custom build UV-impenetrable housing to protect the user against the UV irradiation. If a UVB lamp is not at hand, a UVC lamp, such as for instance found in a UV crosslinker, can be used. Both UVB and UVC irradiation induce 6-4PP and CPD photolesions in DNA that are substrates for NER. However, the amount of damage and tissue penetration of the UV light is different and should be tested experimentally. Also, alternative chemical DNA damaging agents can be used, such as Illudin M (Babu and Schumacher, 2016), cisplatin (Borgermann et al., 2019; Slyskova et al., 2018) or N4QO (Nagao and Sugimura, 1976). However, using chemicals to induce DNA damage often leads to more variation in the results, as chemical treatment requires an incubation time, and can be dependent on cellular uptake and metabolic processing of the chemical. Also, the chemical may need to be removed after application, which will make the assay more laborious.

## Troubleshooting

### Problem 1

The number of (viable) eggs or L1 larvae collected after step 9 of the bleaching protocol is insufficient.

### Potential solution

This can often be simply overcome by bleaching more than one 90 mm NGM plate with gravid adults in step 1 of this protocol. Possibly, the bleaching procedure is too harsh and therefore lethal to the eggs. In this case, the incubation time with NaClO/NaOH in step 5 should be shortened. If still insufficient amounts of viable eggs are collected, it could be considered to skip the bleaching procedure and instead collect synchronized eggs by allowing gravid adult animals to lay eggs on a plate for a short amount of time.

### Problem 2

The data are not reproducible after step 28–29 of the germ cell and embryo UV survival assay or step 36–37 of the L1 larvae UV survival assay.

### Potential solution

(1) Make sure that the UVB lamp is well calibrated and re-calibrate regularly or even replace if necessary. The quality of UV lamps, especially when used often, will diminish over time. Also, always place the worm plates at the same location under the UV lamp, as the UV dose in different areas under the UV lamp may vary.
(2) In the germ cell and embryo UV survival, inconsistent outcomes can be caused by bacterial remnants that shield the animals from UV irradiation. This can be solved by more thorough washing of the worms before UV irradiation. Alternatively, the number of bacteria on the plates could be reduced by (i) reducing the culture growing time of the bacteria either on the NGM plates or in the LB medium, or by (ii) lowering the amount of casein digest in the NGM plates. Another common cause of inconsistent results is that some strains grow slower and therefore have not reached the right developmental stage, i.e., gravid young adult, at the time of irradiation. In this case, it is crucial to test the right time for bleaching a strain, so that worms are young adults when exposed to the UV irradiation.
(3) Make sure that the worms on each plate are equally exposed to the UV irradiation. During the L1 larvae UV survival, the eggs should be plated at the edge outside the bacterial lawn. In our experience, the L1 larvae will then crawl into the food source, which will cause them to be equally exposed to the UV irradiation.

### Problem 3

After step 12 of the germ cell and embryo UV survival assay, animals have not reached adulthood yet.

### Potential solution

As some mutant strains grow slower, it should be checked visually if animals have developed sufficiently to start UV irradiation of their germ cells. If animals are too young, the 72 h incubation time should be increased by irradiating a later time point or bleaching at an earlier time point. Alternatively, a higher incubation temperature than 20°C can be used. Typically, *C. elegans* is cultured between 15°C–25°C and will grow and develop faster at higher temperature.

### Problem 4

After step 33 of the L1 larvae UV survival assay, eggs have not hatched into L1 larvae.

### Potential solution

Some strains show embryonic lethality or develop slower because of which eggs may not hatch sufficiently into L1 larvae. If strains are fragile, this may be exacerbated by the bleaching procedure. This may be overcome by using less harsh bleaching treatment, by shortening the incubation time with NaClO/NaOH in step 5. As alternative to bleaching, synchronized L1 larvae can be achieved by allowing adult animals to lay eggs on plates for a short time.

### Problem 5

Studied gene is essential for worm viability.

### Potential solution

Many genes are essential for organism development and/or reproduction and therefore no viable loss-of-function *C. elegans* mutants for these genes are available in *C. elegans.* An alternative way to study involvement of essential genes in NER using the described protocols is to use RNAi feeding to reduce but not completely knockdown protein levels such that animals are still viable. We have found that this approach works well to perform the UV survival assays.

## Resource availability

### Lead contact

Further information and requests for resources and reagents should be directed to and will be fulfilled by the lead contact, Hannes Lans (w.lans@erasmusmc.nl).

### Materials availability

This study did not generate new unique reagents.

### Data and code availability

This study did not generate any unique datasets or code.

## References

[bib1] Babu V., Schumacher B. (2016). A C. elegans homolog for the UV-hypersensitivity syndrome disease gene UVSSA. DNA Repair (Amst).

[bib2] Bianco J.N., Schumacher B. (2018). MPK-1/ERK pathway regulates DNA damage response during development through DAF-16/FOXO. Nucleic Acids Res..

[bib3] Borgermann N., Ackermann L., Schwertman P., Hendriks I.A., Thijssen K., Liu J.C., Lans H., Nielsen M.L., Mailand N. (2019). SUMO ylation promotes protective responses to DNA -protein crosslinks. EMBO J..

[bib4] Boulton S.J., Gartner A., Reboul J., Vaglio P., Dyson N., Hill D.E., Vidal M. (2002). Combined functional genomic maps of the C. elegans DNA damage response. Science.

[bib5] Brenner S. (1974). The genetics of Caenorhabditis elegans. Genetics.

[bib6] C. elegans Deletion Mutant Consortium (2012). Large-scale screening for targeted knockouts in the Caenorhabditis elegans genome. G3 (Bethesda).

[bib7] Giordano-Santini R., Milstein S., Svrzikapa N., Tu D., Johnsen R., Baillie D., Vidal M., Dupuy D. (2010). An antibiotic selection marker for nematode transgenesis. Nat. Methods.

[bib8] Girard L.R., Fiedler T.J., Harris T.W., Carvalho F., Antoshechkin I., Han M., Sternberg P.W., Stein L.D., Chalfie M. (2007). WormBook : the online review of Caenorhabditis elegans biology. Nucleic Acids Res..

[bib9] Lans H., Hoeijmakers J.H.J., Vermeulen W., Marteijn J.A. (2019). The DNA damage response to transcription stress. Nat. Rev. Mol. Cell Biol..

[bib10] Lans H., Lindvall J.M., Thijssen K., Karambelas A.E., Cupac D., Fensgård O., Jansen G., Hoeijmakers J.H.J., Nilsen H., Vermeulen W. (2013). DNA damage leads to progressive replicative decline but extends the life span of long-lived mutant animals. Cell Death Differ..

[bib11] Lans H., Marteijn J.A., Schumacher B.B., Hoeijmakers J.H.J.J., Jansen G., Vermeulen W. (2010). Involvement of global genome repair, transcription coupled repair, and chromatin remodeling in UV DNA damage response changes during developm. PLoS Genet..

[bib12] Lans H., Vermeulen W. (2011). Nucleotide Excision Repair in Caenorhabditis elegans. Mol. Biol. Int..

[bib13] Lans H., Vermeulen W. (2015). Tissue specific response to DNA damage: C. elegans as role model. DNA Repair (Amst).

[bib14] Nagao M., Sugimura T. (1976). Molecular biology of the carcinogen, 4-nitroquinoline 1-oxide. Adv. Cancer Res..

[bib15] Nance J., Frøkjær-Jensen C. (2019). The Caenorhabditis elegans transgenic toolbox. Genetics.

[bib16] Sabatella M., Thijssen K.L., Davó-Martínez C., Vermeulen W., Lans H. (2021). Tissue-specific DNA repair activity of ERCC-1/XPF-1. Cell Rep..

[bib17] Semple J.I., Garcia-Verdugo R., Lehner B. (2010). Rapid selection of transgenic C. elegans using antibiotic resistance. Nat. Methods.

[bib18] Slyskova J., Sabatella M., Ribeiro-Silva C., Stok C., Theil A.F., Vermeulen W., Lans H. (2018). Base and nucleotide excision repair facilitate resolution of platinum drugs-induced transcription blockage. Nucleic Acids Res..

[bib19] Stiernagle T. (2006). Maintenance of C. elegans. WormBook.

